# Cortical representation of speech temporal information through high gamma-band activity and its temporal modulation

**DOI:** 10.1093/cercor/bhad158

**Published:** 2023-05-11

**Authors:** Shunsuke Tamura, Yoji Hirano

**Affiliations:** Department of Psychiatry, Faculty of Medicine, University of Miyazaki, Miyazaki, Japan; Department of Neuropsychiatry, Graduate School of Medical Sciences, Kyushu University, Fukuoka, Japan; Department of Psychiatry, Faculty of Medicine, University of Miyazaki, Miyazaki, Japan; Department of Neuropsychiatry, Graduate School of Medical Sciences, Kyushu University, Fukuoka, Japan; Institute of Industrial Science, The University of Tokyo, Tokyo, Japan

**Keywords:** speech encoding, high gamma-band activity, temporal envelope, temporal fine structure

## Abstract

Numerous studies have investigated low-frequency (theta-band) and high-frequency (gamma-band) neural activities that are phase-locked to temporal structures, including the temporal envelope and fine structure (TFS) of speech signals. Nonetheless, the neural mechanisms underlying the interaction between envelope and TFS processing remain elusive. Here we examined high gamma-band activities and their low-frequency amplitude modulations while listening to monotone speech (MS) with a fundamental frequency (F0) of 80 Hz and non-speech sounds with similar temporal characteristics to MS, namely an amplitude-modulated click train (AMC). Additionally, we utilized noise-vocoded speech (NVS) to evaluate the impact of eliminating the TFS from MS on the high gamma-band activity. We observed discernible high gamma-band activity at the same frequency as F0 of MS and the train frequency of AMC (80 Hz). Furthermore, source localization analysis revealed that the high gamma-band activities exhibited left hemisphere dominance in both MS and AMC conditions. Finally, high gamma-band activities exhibited amplitude-modulation at the same rate as the stimulus envelope of MS and AMC (5 Hz), though such modulation was not observed in NVS. Our findings indicate that the high gamma-band activity in the left hemisphere is pivotal in the interaction of envelope and TFS information processing, regardless of the nature of the stimulus being speech or non-speech.

## Introduction

The human brain’s ability to accurately and swiftly encode acoustic information is crucial for effective speech communications that demand promptness and interactivity. The high variability of amplitude modulations (AM) in speech sounds requires the auditory system to effectively extract these modulations for optimal speech perception (Arnal et al. 2016). The most distinctive temporal modulation present in the speech waveform transpires at low modulation frequencies (<20 Hz) with a peak rate of 4–6 Hz and is referred to as the “envelope.” In contrast, temporal modulations that occur at a frequency exceeding 50 Hz are defined as the “temporal fine structure” (TFS). Several behavioral studies have demonstrated that a temporal envelope with a modulation rate of ˂10 Hz is sufficient for speech comprehension (Chait et al. 2015; Drullman et al. 1994a, 1994b; Elliott and Theunissen 2009; Ghitza 2011; Shannon et al. 1995). The TFS plays a crucial role in pitch perception and determining sound source localization (Oxenham 2008), and enables the extraction of speech information in noisy situations (Qin and Oxenham 2003; Lorenzi et al. 2006; Moore 2008; Gnansia et al. 2009; Hopkins and Moore 2009; Eaves et al. 2011; Mahajan et al. 2017).

Neural oscillatory activities have garnered substantial attention as a potential mechanism for the encoding of temporal information in speech sounds, both in healthy individuals and those with psychiatric disorders, such as schizophrenia (e.g. Hirano et al. 2020; Poeppel and Assaneo 2020; Hirano and Uhlhaas 2021). Several studies have reported that low-frequency neural oscillatory activity in the delta-band (1–4 Hz) and theta-band (4–8 Hz) is phase-locked to the speech envelope (Assaneo and Poeppel 2018; Boucher et al. 2019; Destoky et al. 2019; Doelling et al. 2014; Etard and Reichenbach 2019; Keitel et al. 2018; Kolozsvári et al. 2021; Ghitza and Greenberg 2009; Ghitza 2011; Park et al. 2015; Peelle et al. 2013; Rimmele et al. 2018; Vanthornhout et al. 2018). Importantly, accurate low-frequency neural tracking of the envelope was observed when the intelligibility of the speech sounds was maintained (Peelle et al. 2013; Etard and Reichenbach 2019). Neural responses to TFS have commonly been measured as a frequency-following response (FFR) or high gamma-band (˃ 80 Hz) activity, which is believed to originate from the bilateral auditory nerve, brainstem inferior colliculus, and bilateral primary auditory cortex (Coffey et al. 2016, 2021). Previous studies have found that the FFR and high gamma-band activity were phase-locked to the fundamental frequency (F0), which is a component of TFS (Anderson et al. 2010, 2011, 2013a, 2013b; Coffey et al. 2016, 2021; Fujihira and Shiraishi 2015; Fujihira et al. 2017; Johnson et al. 2008; Song et al. 2011; Tamura and Sung 2020). Some of these studies have also found a correlation between the neural representation of F0 and its harmonics with speech perception accuracy under noisy conditions (Anderson et al. 2011; Song et al. 2011).

Despite the substantial evidence about the neural mechanism underlying the processing of envelope and TFS information, the interactions between these two factors have yet to be fully understood. This is mainly due to the fact that most studies that have measured FFR and high gamma-band activity have utilized stimuli comprising one or two syllables rather than continuous speech sounds, which are necessary to quantify envelope-driven low-frequency activities. A small number of studies have analyzed high gamma-band activity while subjects were listening to continuous speech sounds and revealed that this activity was modulated in amplitude by speech envelope through temporal response function analysis (Kulasingham et al. 2020; Bachmann et al. 2021). Nevertheless, these studies lacked control conditions, making it impossible to ascertain whether high gamma-band activity was indeed a reflection of TFS information. Specifically, these studies did not employ synthesized speech sounds that lack TFS, such as a noise-vocoded speech (NVS), precluding conformation of the association between high gamma-band activity and F0 entrainment in speech. Additionally, it remains unclear whether the AM of high gamma-band activity related to the envelope is speech-specific. Numerous investigations have examined the similarities and dissimilarities in neural processing between speech and non-speech stimuli, particularly in relation to event-related potentials (ERPs), which typically measures the averaged voltage amplitude at time points of interest after the onset of a stimulus (e.g. Chan et al. 2014; Chen et al. 2021; Näätanen et al. 2001; Tampas et al. 2005). These ERP studies aimed to determine whether speech-evoked neural activity (averaged voltage amplitude) reflects lower-order sensory functions or higher-order functions. However, these studies did not account for specific frequency domains within the context of neural oscillations. Given the nature of human speech sounds that involve a wide range of frequencies, it is crucial to investigate speech-specific activities within the framework of neural oscillations. To the best of our knowledge, there are no studies investigating speech-specificity of multi-band neural oscillatory activity reflecting speech temporal information.

In the present study, we utilized electroencephalography (EEG) to measure high gamma-band activity while subjects listened to a five-syllable word speech sound, which enabled us to evaluate neural oscillatory activity phase-locked to not only F0 but also envelope information. Subsequently, source estimation of F0-related high gamma-band activity was performed, and its low-frequency AM was evaluated. We employed a NVS created from the word speech sound as a control stimulus. We assessed the effect of eliminating TFS from the original speech sound on low-frequency AM of high gamma-band activity by comparing it between original speech and NVS conditions. We hypothesized that if high gamma-band activity and its low-frequency AM, evoked by the original speech sound, reflect interaction of envelope and TFS information processing, such activity would be not detected in the case of the NVS condition. Additionally, a non-speech sound with similar temporal characteristics as the word speech sound was utilized to investigate its speech-specificity. We used discontinuous speech stimuli to comparatively analyze amplitude-modulated high gamma-band activities between speech and non-speech conditions, as temporal characteristics of continuous speech sounds are too highly variable to duplicate them in the non-speech stimulus and it was not feasible to create a continuous non-speech stimulus capable of eliciting high gamma-band oscillatory responses and its theta-rate AM in the same manner as the speech stimulus. We postulated that if interaction of envelope-related and TFS-related neural processing is speech-specific to speech sounds, high gamma-band neural activity would be amplitude-modulated at theta-rate only for original speech condition. Clarifying the mechanisms of neural processing at multiple time scale during speech perception would provide novel insights into interpretation of the conventional model for speech temporal information processing (Poeppel 2003; Poeppel et al. 2008). In addition, comparing speech and non-speech neural processing could reveal whether the interaction of envelope-related and TFS-related neural processing reflects auditory or language processing in the brain.

## Materials and methods

### Participants

The participants were 26 healthy native Japanese speakers (14 males), with a mean age of 37.2 years (23–54 years). All subjects were right-handed [we assessed the handedness of 20 out of 26 participants using an Edinburgh handedness questionnaire (Oldfield 1971)] and had no difficulty listening to the stimuli used in this study. Normal hearing was confirmed by measuring pure tone audiometric thresholds at 500, 1000, and 4000 Hz using an audiometer (AA-58, RION). All subjects were screened using the Structured Clinical Interview (SCID)-non-patient edition, and they or their first-degree relatives did not have an Axis-I psychiatric disorder. The exclusion criteria were as follows: (i) history of neurological illness or major head trauma, (ii) history of electroconvulsive therapy, (iii) history of alcohol/drug dependence or abuse, and (iv) verbal IQ below 75. The study was approved by the Research Ethics Board of the Faculty of Medicine, Kyushu University (approval number: 20192023), and was carried out according to the latest version of the Declaration of Helsinki. All participants gave informed consent before the experiment.

### Experimental design

**Fig. 1:**
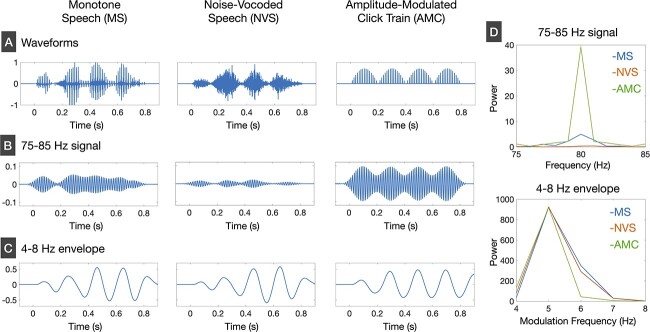
Temporal characteristics of speech and non-speech stimuli. (A) Waveforms of MS, NVS, and the AMC. (B) The band-limited (75–85 Hz) signals of MS, NVS, and AMC. (C) The band-limited (4–8 Hz) envelopes calculated from MS, NVS, and AMC. (D) The spectral power of 75–85 Hz signals and 4–8 Hz envelopes of MS, NVS, and AMC.

We employed three types of stimuli: monotone speech (MS), NVS, and an amplitude-modulated click train (AMC) (Fig. 1). MS had a constant F0 of 80 Hz and its harmonics. The relatively low F0 value was selected to facilitate the measurement of high gamma-band activities phase-locked to high F0 with a high signal-to-noise ratio. The MS was generated through the manipulation of a male Japanese speaker's recorded word speech (“i-chi-bu-bu-n”), procured from the “Spoken Language” section of the DSR Projects Speech Corpus (PASL-DSR) from the Speech Resources Consortium of the National Institute of Informatics (http://research.nii.ac.jp/src/en/PASL-DSR.html). The original speech utterance had an approximate duration of 800 ms. The original speech sound was converted to MS using a pitch-synchronous overlap add (PSOLA) approach in Praat, a speech analysis and synthesis software (Boersma and Weenink 2001). To investigate the impact of TFS elimination from the MS on speech-evoked gamma-band activity and its low-frequency modulation, we employed the NVS. We synthesized the NVS from the MS according to the following steps. First, MS was divided into 33 frequency bands that ranged from 55 to 9657 Hz using an equivalent rectangular bandwidth (ERB)-based auditory filter bank. The boundary frequencies of the band-pass filters (6th-order Butterworth infinite impulse response filter with zero-phase) were defined based on the behavior results concerning the auditory filter shapes (Glasberg and Moore 1990). Second, the signal envelope in each frequency band was extracted using the Hilbert transformation, and the resulting envelope was applied to a zero-phase low-pass filter with a cutoff frequency of 10 Hz (2th-order Butterworth infinite impulse response filter). Finally, the signal envelope extracted for each frequency was multiplied by the band-limited noise at the same frequency band. The AMC was a non-speech sound with a similar temporal profile to the MS. An 80-Hz click train with an 800 ms duration was amplitude-modulated at 5 Hz to create an AMC.

We analyzed the envelope and TFS characteristics of MS, NVS, and AMC. Fig. 1B shows band-pass filtered (75–85 Hz) signal waveforms obtained from the three stimuli (2th-order Butterworth infinite impulse response filter was used for signal filtering). In addition, we calculated the frequency spectrums of these signals by applying a fast-Fourier transform (FFT) to the 75–85 Hz signals. While MS and AMC shared a constant F0 of 80 Hz, AMC had much stronger spectral power at frequencies near 80 Hz than MS. In contrast, the NVS lacked any discernible spectral power at frequencies near 80 Hz (Fig. 1D upper panel). We then analyzed the low-frequency AMs of the three stimuli in the following steps. At first, the original signal was divided into 33 frequency bands using the ERB-based filterbank. Next, the signal envelope in each frequency band was extracted using the Hilbert transformation and was submitted to the band-pass filter of 4–8 Hz. The sum of band-pass filtered envelopes across all frequency bands is displayed in Fig. 1C for each stimulus. The modulation spectrum of 4–8 Hz envelope for each stimulus are shown in Fig. 1D (lower panel). It is confirmed that all the stimuli had strong AM at 5 Hz.

The participants sat in a comfortable chair in a quiet room during the experiment. They were instructed to listen passively to the stimuli with their eyes closed. The stimuli were presented 150 times in each run with an inter-stimulus interval of 1.0 s. All the stimuli were presented to the participants through insert earphones (ER3, Etymotic Research) at a sound pressure level of 80 dBA. The sound pressure level was adjusted using a sound level meter (Type 6240, Aco) and a custom-made coupler (AD-0213, Kyushu InterTech). The presentation order of the three stimuli was determined randomly for each participant. The measurement time for each stimulus was about 4 min. We asked the participants to click a mouse button once per minute to confirm that they were awake. EEG data were recorded using an Active-Two system (BioSemi BV, Amsterdam, Netherlands) with 64 active electrodes on an electrode cap. The EEG data were digitized at 2048 Hz. Electrodes were also placed on the outer canthi and supra-orbit of both eyes to measure eye movements. All channels were referenced to the internal system loop (CMS/DRL electrodes). The data used in this study are not available publicly due to restriction of sharing research data from the Research Ethics Committee but are available on request to a corresponding author.

### E‌EG analysis

The EEG data were analyzed using MNE Python (https://mne. tools/dev/index.html) (Gramfort et al. 2013, 2014). The python codes used for data analysis in this study are available on request to the corresponding author. An offline 1–100 Hz band-pass filter and a notch filter of 60 Hz were applied to the EEG data. Ocular and cardiac artifacts were removed using independent component analysis. Artifact-free EEG data were average referenced, and epochs of 2 s were created. These started at 1 s prior to stimulus onset and lasted until 1 s post-stimulus onset. The numbers of accepted epochs were over 100 for all participants and all stimulus conditions (epochs with peak-to-peak amplitude > 200 μV at any one of the electrodes were rejected). To avoid unfavorable effects of epoch count discrepancy on the outcome of time-frequency analyses, we randomly selected a subset of accepted epochs for further analyses. The selected 100 epochs were averaged across trials separately for each stimulus.

We analyzed the phase-locking factor (PLF) and evoked power on the time-frequency domain (Tallon-Baudry et al. 1996; Roach and Mathalon 2008) to evaluate stimulus-evoked high gamma-band neural activities. The PLF measures the variance of phase across trials on each time-frequency point and ranges from 0 (random distribution) to 1 (perfect phase-locking). For each stimulus condition, the time-frequency PLFs were obtained at all electrodes in time ranges from −1 s to 1 s with 1 ms steps and in frequency ranges from 30 to 100 Hz with 1 Hz steps by applying a Morlet wavelet transform to single trial epochs and calculating the degree of phase variance across epochs for each time-frequency point. The evoked power is the spectro-temporal power of ERPs induced by the external stimulus. The time-frequency evoked powers were obtained for each stimulus condition by applying the Morlet wavelet transforms to averaged waveforms at all electrodes. The wavelet width increased linearly from 2 to 50 cycles from the lowest to the highest frequency.

To identify cortical sources of the high gamma-band neural activities, we firstly constructed whole-brain source space from a template magnetic resonance imaging (MRI) (fsaverage) using FreeSurfer (https://www.freesurfer.net) (Collins et al. 1994) and created the head model with three-layers (scalp, inner skull, and outer skull) using boundary element model. We then used sensor layouts of EEG systems on the fsaverage brain, which are defined by default in MNE-Python. The forward solution of the lead field matrix based on the head model was computed to predict the propagation of electric current from each brain region (mesh-patterned 20,484 vertexes were marked in the brain) to each electrode. Next the inverse solution was obtained using the forward solution and noise covariance matrix calculated using epoch data during the pre-stimulus period (−500 to 0 ms) to estimate brain activity in each brain region from EEG data. For source localization analysis, we adopted a dynamical statistical parametric mapping (dSPM) (Dale et al. 2000; Hämäläinen and Ilmoniemi 1994), which is a method to calculate the activity of the brain using maximum a posteriori estimation. Lastly, the time-frequency PLFs and evoked powers were obtained on each vertex in the brain by transforming them from sensor space to source space separately for each stimulus condition.

The characteristics of low-frequency AM of the high gamma-band activity were evaluated on source space for each stimulus condition as follows. We firstly obtained a time course of frequency (75–85 Hz)-averaged evoked power on the brain regions where gamma-band neural activity phase-locked to F0 was clearly observed [superior temporal sulcus vertical posterior (STSvp) (a part of auditory association cortex) of each hemisphere, which was defined in Human Connectome Project parcellation of the human cortex (Glasser et al. 2016)] (see details in Results section). We further obtained evoked power in the 75–85 Hz frequency band in the bilateral primary auditory cortex (A1). We next applied a 4–8 Hz band-pass filter to the obtained time courses to calculate their low-frequency AM. The power spectrum of the low-frequency AM signal was analyzed using FFT. We lastly evaluated the signal correlation of the low-frequency AM to 4–8 Hz stimulus envelope (Fig. 1C) using cross-correlations analysis, which was often used to examine the relationship between acoustic features of speech sounds and neural activity (e.g. Anderson et al. 2011; Fujihira and Shiraishi 2015; Fujihira et al. 2017; Tamura and Sung 2020). Specifically, the correlation coefficients were calculated by shifting the low-frequency AM signals in time relative to the 4–8 Hz envelope signal to find the shift that produced the maximum correlation. This maximum correlation was defined as the degree of synchronization between the two waveforms. Fisher’s transformation was used to convert the maximum correlation values (Pearson’s *r*) to *z*-scores for parametrical statistical analysis. The AM signals were up-sampled to the sampling frequency of the stimuli (44,100 Hz) before calculating the correlation coefficients.

In addition to the theta-rate AM, we evaluated the spectral power of stimulus-evoked theta-band (4–8 Hz) activity and its signal correlation with the stimulus envelope. The theta-band activities were obtained for each stimulus condition by estimating the regional activity on the area STSvp and A1 in each hemisphere from averaged waveform data at all electrodes using the MNE-dSPM method and applying the 4–8 Hz band-pass filter to the estimated regional activities. For each stimulus condition, the spectral power of the theta-band activity was calculated using FFT. The envelope-phase-locking was also evaluated for each stimulus condition by calculating the cross-correlation between the theta-band activity and 4–8 Hz envelope signal.

### Statistical analysis

For the purpose of conducting statistical analysis on the time-frequency PLF at the electrodes where high PLF values were observed around the 80 Hz range in both MS and AMC conditions, namely the Fz and Pz electrodes, we initially identified the time-frequency areas that showed a significant main effect of stimulus condition by utilizing a cluster-based permutation analysis of variance (ANOVA) (Maris and Oostenveld 2007). The detailed procedure was as follows: First, the *F*-value was calculated using a repeated-measures ANOVA with one within-subject factor including three stimulus conditions for each time-frequency point. Then, the time-frequency areas for which the *F*-value was above that corresponding to a *P*-value ≤ 0.05 were clustered based on temporal and frequency adjacency. Second, cluster-level statistics were calculated by summing the *F*-values within every obtained time-frequency cluster. Third, the cluster-level statistics were tested via empirical distribution of the maximum cluster-level statistics. This distribution was generated by creating a thousand random partitions in the combined time-frequency data across the three conditions and calculating the maximum cluster-level statistic for each partition. Finally, a cluster *P*-value was obtained by comparing the cluster-level statistic of interest against the empirical distribution. The null hypothesis that no difference would exist between the conditions was rejected if the *P*-value ≤ 0.05.

The source distribution of stimulus-evoked high gamma-band activity was estimated for each stimulus condition by calculating the averaged PLF value across time-frequency points where a significant main effect of stimulus condition was observed on sensor space (Fz electrode) at each vertex. In addition, we obtained the regional PLF values from the area STSvp and A1 of both hemispheres for each stimulus condition by calculating the averaged PLF values across vertices in these areas. The obtained regional PLF values were submitted to a two-way repeated-measures ANOVA with stimulus condition and hemisphere as factors for each brain region. In addition, we performed paired *t*-tests between hemispheres separately for each stimulus condition and multiple comparisons with Bonferroni correction between stimulus conditions separately for each hemisphere as post-hoc analyses.

The two-way repeated measure ANOVAs with stimulus condition and hemisphere as factors were performed for 5 Hz power and cross-correlation coefficient, which were obtained from the low-frequency AM on 75–85 Hz evoked power or the theta-band activity, for each brain region. We conducted multiple comparisons with Bonferroni correction between the three stimulus conditions as post-hoc analyses. We lastly calculated Spearman’s correlations of 5 Hz power or cross-correlation coefficient between the low-frequency AM and the theta-band activity for each stimulus condition and hemisphere.

## Results

**Fig. 2:**
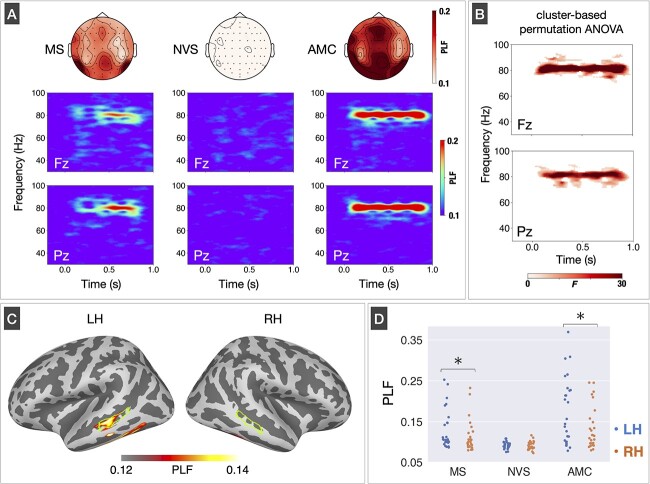
High gamma-band phase-locking factor (PLF) on sensor and source spaces. (A) Mean topographies of high gamma-band (75–85 Hz) PLF during the stimulus period (0–1 s) and mean time-frequency PLF maps at the Fz and Pz electrodes for monotone speech (MS), noise-vocoded speech (NVS), and amplitude-modulated click-train (AMC) conditions. (B) The time-frequency clusters with a significant main effect of stimulus condition at the Fz (top) and Pz electrodes (bottom). For each electrode, the *F*-value at each time-frequency point, calculated through a one-way repeated-measures analysis of variances for cluster formation, is displayed in the time-frequency map. (C) Mean source distribution of high gamma-band PLF for MS condition. The light green lines indicate the areas of superior temporal sulcus vertical posterior (STSvp) in left and right hemispheres (LH and RH). (D) Comparison of high gamma-band PLF values among three stimulus conditions and hemispheres. These values were extracted from the areas of STSvp of both hemispheres, displayed in (A).

The Fz and Pz electrodes had high PLF values in frequency ranges close to F0 of MS and train frequency of AMC as shown in the upper panels of Fig. 2A (topography map of the PLF was calculated by averaging the PLF values across the time-frequency range from 0 to 1 s and from 75 to 85 Hz in each electrode). The time-frequency maps obtained from the Fz and Pz electrodes exhibited elevated PLF values within frequency bands centered at 80 Hz in the MS and AMC conditions, while such activity was not found in the NVS condition (Fig. 2A, lower panel). We applied a cluster-based permutation ANOVA to the time-frequency PLF data and found several time-frequency clusters that had a significant main effect of stimulus condition within the frequency ranges around 80 Hz at the Fz (cluster *P* = 0.001) and Pz electrodes (cluster *P* = 0.001) (Fig. 2B).

We next attempted to identify the signal sources of TFS-related high gamma-band activities by transforming time-frequency PLFs from the sensor to source spaces. The source distribution of high gamma-band PLF in the MS condition is illustrated in Fig. 2C. The high gamma-band activity, which reflects F0 information, was predominantly detected within the area STSvp of the left hemisphere. We next extracted individual PLF values from the area STSvp of both hemispheres separately for each stimulus condition (Fig. 2C, light green areas) and compared them between stimulus conditions and hemispheres (Fig. 2D). The two-way repeated-measures ANOVA revealed significant main effects of stimulus condition [*F*(2,50) = 26.79, *P* < 0.001] and hemisphere [*F*(1,25) = 6.47, *P* = 0.018]. The significant interaction between stimulus condition and hemisphere was also significant [*F*(2,50) = 6.84, *P* = 0.002]. The post-hoc analyses revealed a statistically significant increase in PLF values within the LH compared to the right hemisphere (RH) during both the MS (*P* = 0.028) and AMC conditions (*P* = 0.013). However, no significant difference between hemispheres was found in the NVS condition (*P* = 0.69). In addition, the multiple comparisons between stimulus conditions showed that the PLF was significantly higher for AMC condition compared to MS and NVS conditions in both LH (AMC vs. MS: *P* < 0.001, AMC vs. NVS: *P* < 0.001) and RH (AMC vs. MS: *P* < 0.001, AMC vs. NVS: *P* < 0.001). We also found a significantly increased PLF for MS condition than NVS condition in both hemispheres (left: *P* = 0.005; right: *P* = 0.043).

**Fig. 3:**
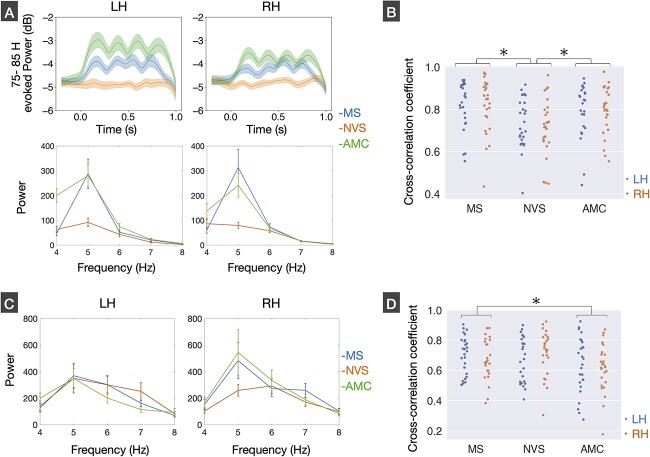
Modulation characteristic of high gamma-band and theta-band activities. (A) Mean time courses of 75–85 Hz evoked power in the areas of superior temporal sulcus vertical posterior (STSvp) of left and right hemispheres (LH and RH) for monotone speech (MS), noise-vocoded speech (NVS), and amplitude-modulated click train (AMC) conditions. The power spectrum of low-frequency (4–8 Hz) AM of 75–85 Hz evoked power for each stimulus condition and each hemisphere. (B) Comparisons of signal correlation (cross-correlation coefficient) of the low-frequency amplitude modulated (AM) signal with stimulus envelope between stimulus conditions and hemispheres. (C) The power spectrum of stimulus-evoked theta-band (4–8 Hz) activity in the area of STSvp for each stimulus condition and each hemisphere. (D) Comparisons of signal correlation (cross-correlation coefficient) of the theta-band activity with stimulus envelope between stimulus conditions and hemispheres.

The temporal progression of evoked power within the 75–85 Hz range in the area STSvp was illustrated in the upper panel of Fig. 3A, separately for each stimulus condition and each hemisphere. While the temporal fluctuations of 75–85 Hz evoked power were markedly evident during both the MS and AMC conditions, there were no such fluctuations during the NVS condition. We then obtained the low-frequency AM by applying a band-pass filter of 4–8 Hz to the extracted time course, and subsequently calculated its spectral power separately for each stimulus condition and hemisphere. Our findings indicate that the low-frequency AMs of TFS-related high gamma-band activities in both hemispheres exhibited the highest power at the peak modulation frequency of the stimulus envelope (5 Hz) for both the MS and AMC conditions, as evidenced by the bottom panels in Fig. 3A. The main effect of the stimulus condition was confirmed by two-way repeated-measures ANOVA on the 5 Hz power [*F*(2,50) = 10.62, *P* < 0.001]. There were no significant main effect of hemisphere [*F*(1,25) = 0.078, *P* = 0.78] and interaction between stimulus condition and hemisphere [*F*(2,50) = 0.51, *P* = 0.61]. The multiple comparisons between stimulus conditions revealed that the 5 Hz powers in the MS and AMC conditions were significantly higher than that in the NVS condition (MS vs. NVS: *P* = 0.004, AMC vs. NVS: *P* < 0.001), while no significant difference between the MS and AMC conditions was found (*P* = 1.00).

In addition, we analyzed signal correlation of the low-frequency AM with the stimulus envelope (Fig. 1C) separately for each stimulus condition. Fig. 3B shows comparisons of cross-correlation coefficients between stimulus conditions and hemispheres. The two-way repeated-measures ANOVA showed a significant main effect of stimulus condition [*F*(2,50) = 8.80, *P* < 0.001], while there were no significant main effect of hemisphere [*F*(1,25) = 0.43, *P* = 0.52] and interaction of stimulus condition and hemisphere [*F*(2,50) = 0.08, *P* = 0.93]. The post-hoc analyses revealed that the cross-correlation coefficients in the MS and AMC conditions were significantly higher than those in the NVS condition (MS vs. NVS: *P* = 0.001, AMC vs. NVS: *P* = 0.029). There was no significant difference between the MS and AMC conditions (*P* = 0.88).

Finally, we evaluated the characteristics of theta-band activity on the area STSvp of both hemispheres and examined their relationships with those of the low-frequency AM of high gamma-band activity. Fig. 3C shows comparison of theta-band power spectrums between stimulus conditions separately for each hemisphere. When a two-way repeated-measures ANOVA was conducted on 5 Hz power, no significant main effects [stimulus condition: *F*(2,50) = 1.07, *P* = 0.35; hemisphere: *F*(1,25) = 0.50, *P* = 0.49] or interaction [*F*(2,50) = 0.94, *P* = 0.40] were found. The cross-correlation coefficients between theta-band activity and stimulus envelope were also compared between stimulus conditions and hemispheres (Fig. 3D). We conducted two-way repeated measure ANOVAs and found a significant main effect of stimulus condition [*F*(2,50) = 3.84, *P* = 0.028]. However, there were no significant main effect of hemisphere [*F*(1,25) = 0.02, *P* = 0.89] or interactions between the two factors [*F*(2,50) = 0.88, *P* = 0.42]. The post-hoc analysis revealed higher cross-correlation in the MS condition compared to AMC condition (*P* = 0.049), while there were no significant differences in the other combinations (MS vs. NVS: *P* = 1.00; NVS vs. AMC: *P* = 0.17). In summary, the pattern of theta-band activity differed significantly from that of the low-frequency AM of TFS-related high gamma-band activity. Additionally, we examined the correlations of 5 Hz power and cross-correlation coefficients of theta-band activity with those of the low-frequency AM, separately for each stimulus condition. However, we did not observe significant correlations for either 5 Hz power (MS left: *rho* = −0.06, *P* = 0.77, MS right: *rho* = 0.03, *P* = 0.88; NVS left: *rho* = 0.33, *P* = 0.10; NVS right: *rho* = −0.27, *P* = 0.19; AMC left: *rho* = 0.00, *P* = 0.99; AMC right: *rho* = −0.04, *P* = 0.84) or cross-correlation coefficient (MS left: *rho* = −0.22, *P* = 0.28, MS right: *rho* = 0.21, *P* = 0.30; NVS left: *rho* = −0.06, *P* = 0.77; NVS right: *rho* = −0.36, *P* = 0.07; AMC left: *rho* = −0.22, *P* = 0.28; AMC right: *rho* = −0.03, *P* = 0.88) for any stimulus condition.

We show detailed results of the stimulus-evoked high gamma-band activity and its theta-rate AM on bilateral A1 in Supplementary Materials. In summary, we found high gamma-band activity in bilateral primary cortex during both MS and AMC stimulations, without any hemispheric laterality (Supplementary Result S1). In addition, there were no significant differences in the theta-rate AM among the three stimulus conditions (Supplementary Result S2).

## Discussion

We measured neural oscillatory activities using MS created from five-syllable word sound to investigate neural mechanisms underlying speech perception, providing a novel perspective distinct from prior studies that primarily focused on either envelope-related or TFS (F0)-related neural activity. The EEG source localization analysis revealed that the left STS predominantly exhibited high gamma-band activities in responses to speech and non-speech stimuli containing TFS information, specifically MS and AMC. In addition, we discovered that the high gamma-band activity associated with TFS was amplitude-modulated at a frequency identical to the stimulus envelope (5 Hz) in this region, regardless of whether the auditory input was speech or non-speech. We will discuss these findings below in more detail.

We observed high gamma-band activities evoked by the stimuli at the same frequency as F0 of MS and train frequency of AMC (80 Hz), whereas the NVS stimulus did not evoke such activities, as depicted in Fig. 2A. Based on these findings, the presence of stimulus-evoked high gamma-band activity during speech perception may heavily rely on the availability of TFS information. Although Kulasingham et al. (2020) suggested that high gamma-band activities elicited by continuous speech sounds were dominantly driven by the stimulus envelope, with a lesser contribution from TFS, they did not compare high gamma-band activities between speech stimuli with and without TFS information. The source localization analysis of TFS-related high gamma-band activity suggested that the prominent signal source for the MS condition could be estimated within the left STSvp (Fig. 2C). Furthermore, our investigation revealed that TFS-related high gamma-band activities exhibited prominent left hemisphere dominance in both speech (MS) and non-speech (AMC) conditions (Fig. 2D). In contrast to our present findings, several previous studies have shown that high gamma-band activity phase-locked to F0 of speech sounds and 80 Hz steady-state stimulation produced right hemisphere dominance (Coffey et al. 2021; Isomura et al. 2016; Oda et al. 2012; Ross et al. 2020; Tsuchimoto et al. 2011). The underlying reasons for the discrepancy between our study and previous studies remain uncertain; however, one possible explanation is that we employed stimuli that had an oscillatory envelope, while the stimulus used in the previous studies were single-syllable sounds or an 80 Hz click train lacking an oscillatory envelope.

As a candidate of the neural mechanism underlying the interaction of envelope and TFS information processing, we found a low-frequency AM of TFS-related high gamma-band activities at the same modulation frequency as stimulus envelope during the MS and AMC conditions (Fig. 3A upper panel). Furthermore, we observed that the degree of its envelope-phase-locking was higher for MS and AMC conditions compared to NVS condition (Fig. 3B). These results indicate that theta-rate AM of high gamma-band activity was precisely phase-locked to speech envelope only when the auditory stimulus contains TFS information and that such a phase-locking was observed in common with high gamma-band activity elicited by non-speech sound. In other words, the occurrence of TFS-related high gamma-band activity is assumed to be essential for connecting TFS and envelope information regardless of whether auditory input was speech or non-speech. We subsequently compared the resultant pattern of stimulus-evoked theta-band activity with the low-frequency AM of high gamma-band activity. Our findings revealed that theta-band activity exhibited a peak power of 5 Hz in all stimulus conditions (Fig. 3C lower panel). Furthermore, we observed significant signal correlation between theta-band activity with stimulus envelope in all stimulus conditions (Fig. 3D). These results suggest that theta-band activity reflects stimulus envelope information regardless of whether the auditory input contains TFS information, whereas the low-frequency AM of TFS-related high gamma-band activity heavily relies on TFS information. Additionally, we failed to identify any significant correlations between the theta-band activity and the low-frequency AM regarding the 5 Hz power and envelope-phase-locking. Taken together, it is conceivable that the low-frequency (theta-rate) AM of the TFS-related high gamma-band activity occurs independently of the envelope-driven theta-band activity.

We investigated the role of speech-evoked high gamma-band activity, which may encode both envelope and TFS information. Our present study considers the perspective of a well-known oscillatory multi-time resolution model of speech perception proposed by Poeppel (Poeppel 2003; Poeppel et al. 2008). According to this model, the right auditory cortex preferentially processes the slow temporal envelope of speech sounds via theta-band activity, whereas the left auditory cortex is sensitive to the TFS via low-gamma band (25–50 Hz) activity. This model has been supported by several experimental and computational studies that have examined the functional role of cross-frequency coupling between theta-band and low-gamma-band activity in speech perception (Gross et al. 2013; Hyafil et al. 2015; Lizarazu et al. 2019). However, based on our findings, we propose that amplitude-modulated high gamma-band activities in the left auditory-related areas are also critical for the interaction between TFS and envelope processing. Thus, to fully understand the oscillatory mechanisms underlying the perceptual processing of speech temporal information, it is necessary to examine not only low-gamma but also high-gamma activities.

The perceptual processing of envelope and TFS information via high gamma-band activity within the left STSvp was commonly observed for both speech (MS) and non-speech conditions (AMC). This finding suggests that the left posterior STS plays an essential role in processing auditory temporal information, irrespective of whether the auditory input is speech or non-speech. A previous fMRI study has also demonstrated commonalities between speech and non-speech processing in the left posterior STS using a sine-wave speech, which can be perceived as speech or non-speech depending on the listeners’ auditory experience. This study suggested that this region may be a prerequisite for perceiving sounds as speech (Möttönen et al. 2006). There is a limited number of studies examining the similarities and differences in oscillatory neural activities in responses to speech and non-speech sounds (Morillon et al. 2010; Di Liberto et al. 2015). Therefore, the present findings could provide valuable insights into the neural specificity of speech in terms of its oscillatory characteristics.

Although our study provided meaningful evidence regarding the neural mechanisms underlying auditory processing of speech temporal information, there are several limitations that need to be addressed. Firstly, the relatively low F0 of MS (80 Hz) may have affected the results, given that voiced speech sounds uttered by Japanese men and women have F0 values of 80–200 Hz and 160–360 Hz, respectively (Graham 2014). In addition, using MS with a constant F0 to measure stimulus-evoked high gamma-band activity may have introduced unnatural qualities that could have impacted our results. This is because the temporal variation of F0 is known to contribute to speech perception, particularly in noisy environments (Shen and Souza 2017; Wu 2019). Secondly, presenting discontinuous speech sounds repetitively to the participants is highly unnatural and demotivating, which may have affected their neural responses. Recent technical advancements in EEG/MEG data analysis have enabled to analyze neural activities when a participant listens to continuous natural speech without repetition (e.g. Brodbeck and Simon 2020; Gillis et al. 2022). Taken together, future studies should investigate how to measure amplitude-modulated high gamma-band activity in responses to the continuous speech and non-speech sounds to confirm whether our findings can be generalized to speech perception and comprehension in daily life. Then, as we only measured neural activities in passive listening conditions, we were unable to discuss the contribution of high gamma-band activity and its low-frequency AM to speech intelligibility and comprehension. Therefore, future studies must investigate the effects of different listening conditions on neural responses. Finally, we computed the signal sources of TFS-related high gamma-band activity using EEG, which is constrained in its source localization capabilities. Considering the possibility that variations in source estimation precision between EEG and MEG could result in inconsistencies among studies, further investigation using MEG with a higher spatial resolution (Hämäläinen et al. 1993) is needed to confirm the reliability of our findings.

In summary, the current investigation has revealed several important characteristics of high gamma-band activities during speech perception. Most importantly, our findings propose that the stimulus-evoked high gamma-band activity and its AM on the left STS processes both of envelope and TFS information, regardless of the nature of the acoustic input (speech or non-speech). Thus, the left STS plays an essential role in processing auditory temporal information critical for speech perception via high gamma-band activity and its temporal modulation. It is plausible that these findings will lead to a more advanced and comprehensive understanding of the oscillatory mechanisms that underlie speech perception in the brain. Moreover, our experimental design that provided new insights into auditory- and speech-related neural oscillatory activity might be of use in elucidating the pathophysiological mechanisms in individuals with schizophrenia (Hirano and Uhlhaas 2021; Tamura et al. 2022). Despite the considerable number of investigations highlighting an impaired gamma-band oscillatory activity during auditory-steady-state response stimulation (Thuné et al. 2016; Onitsuka et al. 2022), only a limited number of studies have utilized speech sounds to assess gamma-band oscillatory dysfunctions in schizophrenia. It is expected that assessing speech-evoked gamma-band activity in patients with schizophrenia would clarify neural mechanisms underlying language dysfunctions that are unique to this disorder (Meyer et al. 2021).

## Supplementary Material

SUPPLEMENTARY_bhad158
