# Supplementary material for: Cortical representation of speech temporal information through high gamma-band activity and its temporal modulation
Source: Cereb Cortex. 2023 May 11;33(13):8773–82. doi: 10.1093/cercor/bhad158 (PMC10321101; doi:10.1093/cercor/bhad158)
Supplement: SUPPLEMENTARY_bhad158 [file supplementary_bhad158.zip › SUPPLEMENTARY_bhad158.docx]

**Supplementary Material**

**Supplementary Results 1:**

We extracted individual PLF values from bilateral A1 separately for each stimulus condition and compared them between stimulus conditions and hemispheres (**Supplementary Figure 1**). The two-way repeated measures ANOVA revealed a significant main effect of stimulus condition (*F*(2,50) = 14.34, *p* < 0.001), although a main effect of hemisphere (*F*(1,25) = 1.27, *p* = 0.27) and a significant interaction between stimulus condition and hemisphere (*F*(2,50) = 2.02, *p* = 0.16) were not significant. Multiple comparisons between stimulus conditions revealed a significant increase in the PLF for AMC condition in comparison to MS and NVS conditions (AMC vs. MS: *p* = 0.049, AMC vs. NVS: *p* < 0.001). We also found a significant increase in the PLF for MS condition as compared to NVS condition in both hemispheres (*p* = 0.016).

**Supplementary Results 2:**

The temporal progression of evoked power within the 75-85 Hz range in A1 was illustrated in the upper panel of **Supplementary Figure 2A**, separately for each stimulus condition and each hemisphere. We then obtained the low-frequency AM by applying a bandpass filter of 4-8 Hz to the extracted time course, and subsequently calculated its spectral power separately for each stimulus condition and hemisphere. Our findings indicate that the low-frequency AMs of TFS-related high gamma-band activities in both hemispheres exhibited the highest power at the peak modulation frequency of the stimulus envelope (5 Hz) for both the MS and AMC conditions (bottom panels in **Supplementary Figure 2A**). The main effect of the stimulus condition was confirmed by two-way repeated measures ANOVA on the 5 Hz power (*F*(2,50) = 6.49, *p* = 0.006). There were no significant main effect of hemisphere (*F*(1,25) = 0.09, *p* = 0.77) and interaction between stimulus condition and hemisphere (*F*(2,50) = 0.52, *p* = 0.60). The multiple comparisons between stimulus conditions revealed that the 5 Hz power in the MS condition was significantly higher than that in the NVS condition (*p* = 0.04), while no significant differences were observed in the other combinations. In addition, we analyzed signal correlation of the low-frequency AM with the stimulus envelope separately for each stimulus condition. **Supplementary Figure 2B** shows comparisons of cross-correlation coefficients between stimulus conditions and hemispheres. There were no significant main effects of stimulus condition (*F*(2,50) = 1.67, *p* = 0.21) and hemisphere (*F*(1,25) = 0.46, *p* = 0.50). Additionally, the interaction between stimulus condition and hemisphere was not significant (*F*(2,50) = 0.21, *p* = 0.81).


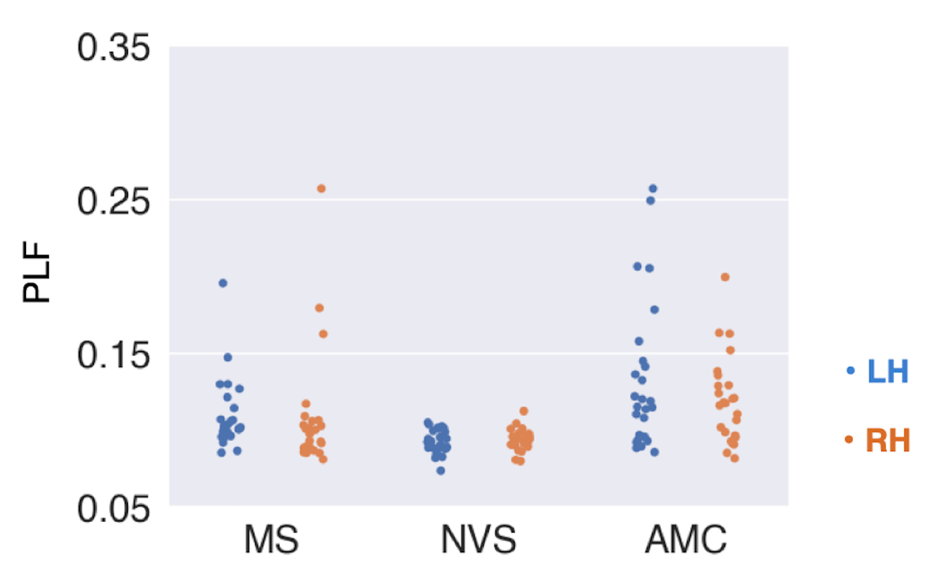


**Supplementary Figure 1.**

**High gamma-band phase-locking factor (PLF) on primary auditory cortex (A1).**

Comparison of high gamma-band PLF values among three stimulus conditions (monotone speech [MS], noise-vocoded speech [NVS], and amplitude-modulated click train [AMC]) conditions) and hemispheres (LH and RH). These values were extracted from the areas of bilateral A1.


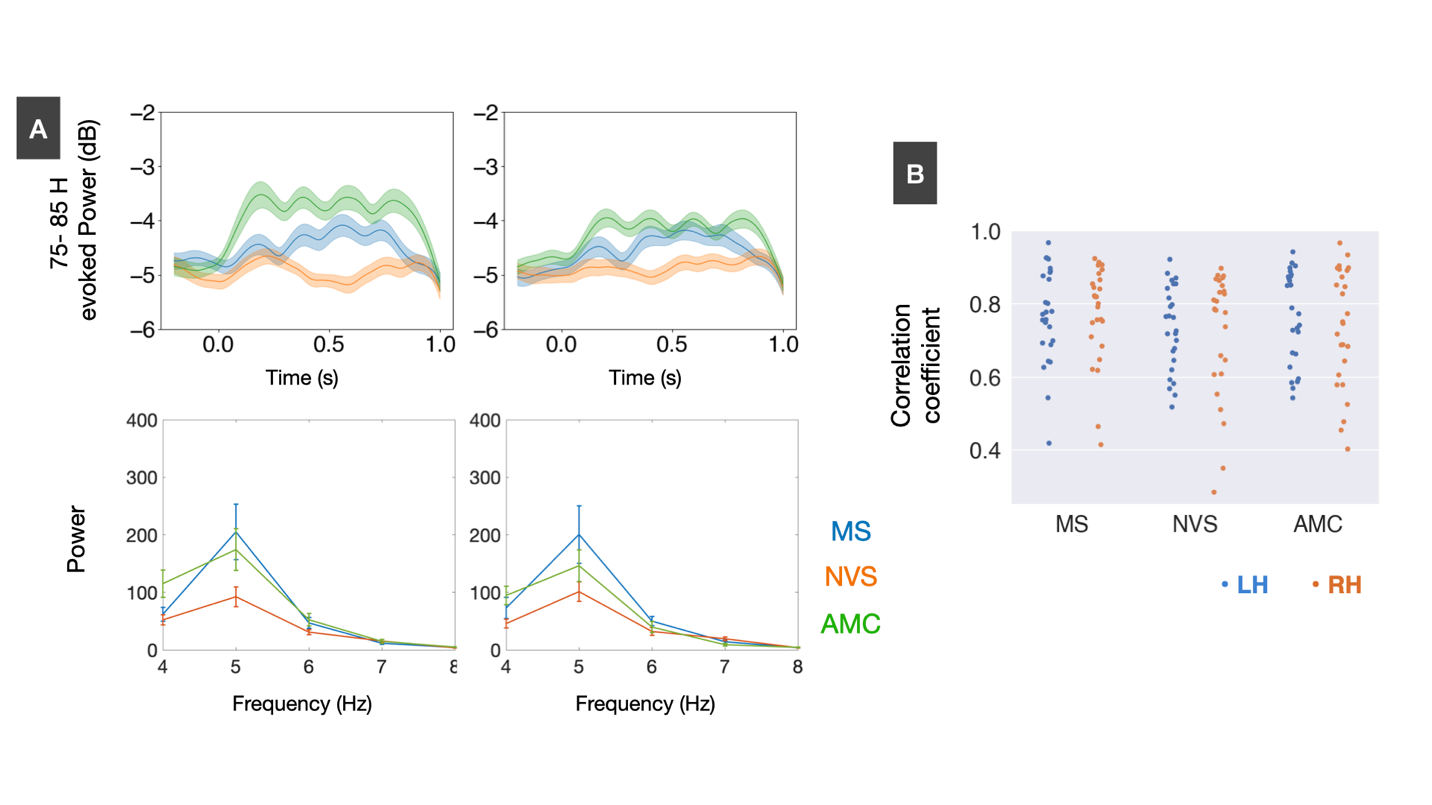


**Supplementary Figure 2.**

**Modulation characteristic of TFS-related high gamma-band activity.**

**A:** Mean time courses of 75-85 Hz evoked power in the primary auditory cortex of left and right hemispheres (LH and RH) for monotone speech (MS), noise-vocoded speech (NVS), and amplitude-modulated click train (AMC) conditions. The power spectrum of low-frequency (4-8 Hz) amplitude modulation (AM) of 75-85 Hz evoked power for each stimulus condition and each hemisphere. **B:** Comparisons of signal correlation (cross-correlation coefficient) of the low-frequency AM signal with stimulus envelope between stimulus conditions and hemispheres.
